# Epigenetic Regulation and Gut Microbiota Dysbiosis in Age-Related Macular Degeneration

**DOI:** 10.3390/genes17101219

**Published:** 2026-10-01

**Authors:** Stamatios Lampsas, Chrysa Agapitou, Gerasimia-Marina Chardalia, Dimitrios Soulimiotis, Konstantinos Papastamopoulos, Panagiotis Theodossiadis, Irini Chatziralli

**Affiliations:** 2nd Department of Ophthalmology, School of Medicine, National and Kapodistrian University of Athens, Attikon University Hospital, 1, Rimini Street, Haidari, 12462 Athens, Greece; stamlampsas@med.uoa.gr (S.L.); chr.agapitou@gmail.com (C.A.); gerasimiamarina@gmail.com (G.-M.C.); jim7soul02@gmail.com (D.S.); papastamk@gmail.com (K.P.); patheo@med.uoa.gr (P.T.)

**Keywords:** age-related macular degeneration, pathogenesis, epigenetics, gut-retina axis, gut microbiota

## Abstract

Age-related macular degeneration (AMD) is a leading cause of visual impairment. Beyond the classical pathogenetic pathways, increasing evidence suggests that epigenetic regulation and the gut–retina axis may substantially contribute to AMD development and progression. This review synthesizes current evidence on two increasingly recognized and interconnected domains of AMD pathogenesis: epigenetic regulation and gut microbiota dysbiosis. Epigenetic processes, such as DNA methylation, histone modifications, and non-coding RNAs, can influence gene function involved in oxidative stress responses, complement regulation, angiogenesis, and inflammation, while several related markers have emerged as potential diagnostic and prognostic biomarkers. Moreover, growing evidence suggests a functional gut–retina axis, in which alterations in the gut microbiota impair intestinal barrier function and facilitate the systemic passage of bacteria and microbial metabolites, potentially affecting retinal inflammation and immune responses. Furthermore, several microbial metabolites, including short-chain fatty acids, lipopolysaccharide, bile acids, tryptophan derivatives, and trimethylamine-N-oxide, may have either protective or harmful effects on retinal and choroidal homeostasis, with altered circulating and fecal concentrations reported in patients with AMD compared with controls. These findings support AMD as a systemically and molecularly interconnected disorder, while further longitudinal, mechanistic, and multi-omics studies are required to establish causality, define interactions between epigenetic and microbiome-related pathways, and clarify their potential utility as biomarkers and therapeutic targets.

## 1. Introduction

Age-related macular degeneration (AMD) is a chronic, progressive degenerative disease and a major cause of visual impairment among individuals aged 55 years and older [1]. AMD has two distinct subtypes: Dry AMD, which mainly involves progressive retinal pigment epithelium degeneration and adjacent photoreceptor cells, causing their gradual loss, and Wet AMD, which results from abnormal blood vessel growth originating in the choroid—a process known as choroidal neovascularization (CNV)—which invades the retinal layers and disrupts normal function [2]. The two subtypes are not entirely separate, but they converge on shared mechanisms.

Traditional mechanisms include oxidative microvascular damage, chronic low-grade inflammation, dysregulated complement activity, accumulation of lipid deposits, and, for the neovascular subtype, angiogenesis driven by vascular endothelial growth factor (VEGF) [1,3]. Increasing attention has focused on mechanisms associated with the retinal pigment epithelium (RPE)–Bruch’s membrane–choriocapillaris complex that plays a central role in AMD pathogenesis [4,5].

Beyond these pathogenic mechanisms, emerging evidence has raised additional molecular and cellular pathways that may contribute to AMD development and progression. Mitochondrial dysfunction has been recognized as an upstream contributor to AMD. Impaired mitochondrial quality control can lead to ATP depletion, mitochondrial DNA damage, and release of damage-associated signals, thereby promoting inflammation, complement activation, and reduced RPE resilience [4,6].

Complement involvement in AMD appears to reflect an imbalance between local activation and regulation within the outer retina and choroid, rather than nonspecific pathway activation alone. Persistent complement signaling may shift physiological immune surveillance toward chronic inflammation, while interacting with oxidative stress, mitochondrial dysfunction, lipid metabolism, and immune-cell activation. These findings position complement dysregulation within a broader interconnected pathogenic network in AMD [7,8].

Single-cell and single-nucleus sequencing has revealed substantial cellular heterogeneity within the retina, RPE, and choroid, with RPE, endothelial, fibroblast, macrophage, and immune-cell populations each showing region- and disease-specific transcriptional signatures. This indicates that AMD pathology cannot be attributed to any single cell type but instead reflects complex intercellular communication networks—particularly between choroidal endothelial cells and infiltrating immune cells during neovascularization. Integration of these transcriptomic datasets with genetic data has further begun to map AMD-associated risk variants onto specific cell populations and regulatory pathways, linking genetic susceptibility to cellular dysfunction [9,10,11,12].

A related mechanism involves partial epithelial–mesenchymal transition (EMT) in RPE cells under chronic oxidative, inflammatory, or hypoxic stress, marked by loss of epithelial character, migratory behavior, and extracellular matrix remodeling. This process is implicated in advanced AMD, contributing to RPE dysfunction, subretinal fibrosis, and neovascular lesion progression, and is regulated by pathways already tied to AMD pathogenesis—including Transforming growth factor beta (TGF-β), Hypoxia-inducible factor (HIF) signaling, mitochondrial dysfunction, autophagy, inflammation, complement activation, and epigenetic regulation—underscoring the convergence of these seemingly distinct disease processes [13].

These findings suggest that AMD pathogenesis extends not only to classical mechanisms but also encompasses a broader network of molecular and environmental regulators. The present review aims to synthesize current evidence on epigenetic regulation and gut microbiota dysbiosis as interconnected contributors to AMD pathogenesis.

## 2. Literature Search Strategy

A literature search was conducted to identify studies addressing epigenetic regulation, gut microbiota dysbiosis, and microbiota-derived metabolites in age-related macular degeneration. The literature search was updated through September 2026. PubMed was the principal database searched, using combinations of terms including “age-related macular degeneration” OR “AMD” with “epigenetics,” “DNA methylation,” “histone modification,” “microRNA,” “non-coding RNA,” “gut microbiota,” “gut microbiome,” “gut–retina axis,” “short-chain fatty acids,” “lipopolysaccharide,” “bile acids,” “tryptophan metabolites,” and “trimethylamine-N-oxide”. Human observational and molecular studies, postmortem retinal/RPE studies, experimental animal models, in vitro studies, bioinformatic and multi-omics analyses were considered when they contributed directly to the biological mechanisms discussed. Reference lists of key articles were also manually screened to identify additional relevant publications.

## 3. Epigenetic Regulation

Epigenetics encompasses both heritable and environmentally induced mechanisms that modify chromatin structure and gene expression, without altering the underlying DNA sequence [14,15]. Epigenetic dysregulation has been implicated in the pathogenesis of numerous human diseases [16], including ocular pathologies [17]. Notably, over the last few decades, as the prevalence of AMD appears to be increasing [18] and the necessity for novel therapies has arisen, researchers have begun exploring a potential linkage between epigenetics and AMD pathophysiology [19,20,21]. Epigenetic mechanisms are influenced by environmental factors such as smoking, alcohol use, metabolic syndrome, and physical activity [22]. These factors often induce oxidative stress, chronic inflammation, and interfere with lipid metabolic mechanisms and inflammatory cytokine signaling, all of which are implicated in AMD pathogenesis [23,24].

### 3.1. DNA Methylation

One of the primary and most studied epigenetic mechanisms is DNA methylation. The mechanism entails the covalent transfer of a methyl group to the fifth carbon of a cytosine residue, resulting in the formation of 5mC, mostly in CpG sites across the genome. Following methylation, especially in gene promoter regions, transcription is usually repressed, resulting in gene silencing or reduced gene expression. DNA methylation is catalyzed by enzymes called DNA methyltransferases (DNMTs). There are three principal types of DNMTs: DNMT1, DNMT3a, and DNMT3b. DNMT1, the maintenance DNMT, plays a key role in DNA replication by preserving the methylation patterns during DNA strand copying. DNMT3a and DNMT3b, also called de novo DNMTs, establish new methylation patterns [25]. Growing evidence indicates that DNA methylation is a contributing factor in AMD pathophysiology, through regulating genes involved in oxidative stress, angiogenesis, complement pathways, inflammation, and extracellular matrix deposition [26,27,28].

Retinal function is highly dependent on oxygen supply, as the visual cycle is a highly energy-demanding process [29]. During this energy consumption, reactive oxygen species (ROS) are naturally produced. ROS are known to induce oxidative stress, but the retina possesses antioxidant mechanisms in order to compensate for and preserve its homeostasis. However, if these mechanisms are insufficient or dysregulated, oxidative stress accumulates, and retinal tissue dysfunction occurs [30,31]. AMD represents a multifactorial pathology, characterized by a positive feedback loop between ROS and inflammation [32]. Epigenetic repression of genes involved in the ROS detoxification process may lead to a hypofunctional retinal defense system and accumulation of oxidative injury in RPE cells [33].

Among genes involved in the antioxidant defense, members of the glutathione S-transferase mu (GSTM) family have emerged as principal targets of DNA methylation alterations in AMD. Hunter et al. analyzed postmortem RPE/choroidal and neurosensory retinal samples of AMD patients and compared them to age-matched controls [34]. Notably, they identified hypermethylation of the GSTM1 promoter, along with reduced levels of mRNA transcripts of both GSTM1 and GSTM5 in AMD patients. Moreover, they detected reduced protein expression, specifically in the RPE/choroid. As GSTMs catalyze glutathione-dependent detoxification reactions, their decreased expression in retinal tissues may impair the clearance of lipid peroxidation residues, as well as reactive electrophiles, thereby enhancing oxidative injury to the outer retina [35,36].

More recently, Advani et al. integrated retinal DNA methylation, gene expression, and genotype data from 160 postmortem human retinal samples, identifying 87 gene-epigenome interactions through methylation quantitative trait loci (mQTLs) and expression quantitative trait loci (eQTLs) [27]. Among genes implicated in oxidative stress pathways, PARK7 was associated with five retinal mQTLs and one eQTL, indicating that local genetic variation may influence both CpG methylation and PARK7 expression in the retina. PARK7 encodes DJ-1, a multifunctional protein that plays a neuroprotective role against oxidative stress-induced cell death [37]. Consistent with this biological role, Bonilha et al. demonstrated that PARK7-deficient mice displayed morphological retinal abnormalities, altered electroretinogram (ERG) findings, and increased oxidative stress [38]. However, these observations warrant cautious interpretation, as they reflect genetically regulated methylation and PARK7 expression rather than direct AMD-specific differential methylation. Therefore, PARK7 should be regarded as a candidate epigenetically regulated gene rather than a validated methylation marker of AMD.

Persistent inflammation and complement dysregulation also constitute central mechanisms in the pathophysiology of AMD [39,40]. Interleukin-17 (IL-17) is the primary cytokine produced by Th17 cells, inducing pro-inflammatory responses. More specifically, IL-17A promotes inflammasome activation in the macula and RPE cell toxicity in patients with AMD [41]. Notably, serum samples from human subjects with AMD exhibited significantly higher IL-17 concentrations compared to controls [42]. Furthermore, Wei et al. found that the IL-17RC gene promoter was hypomethylated in 202 AMD patients, with no significant differences detected in the methylation patterns between wet and dry AMD [43]. They also showed that IL-17RC hypomethylation increased IL-17RC protein expression in peripheral blood cells, rendering it a potential serum biomarker for AMD. Moreover, Liang et al. conducted integrated methylation and expression analyses, identifying differential methylation patterns in immune-related genes such as HLA-A, HLA-B, and HLA-DRB1, and characterized them as hub genes for both extramacular and macular AMD [44]. In addition, pertaining to the complement activation mechanism, complement factor H (CFH) is a known inhibitor of the alternative complement pathway, and particular mutations in the CFH gene are considered high risk for AMD pathology. CpG methylation of the CFH promoter has been linked to downregulation of CFH protein in RPE cells from AMD patients, which leads to complement activation [45].

Hypoxia has been considered a major risk factor in the pathogenesis of AMD, especially during choroidal neovascularization seen in wet AMD. Impaired choroidal perfusion and progressive loss of the choriocapillaris lead to permanent oxidative stress in RPE cells, which results in angiogenic signaling [46]. DNA methylation has been proposed as a potential mechanism regulating the expression of genes implicated in hypoxia sensing and angiogenic pathways in the retina. However, evidence remains limited. Hypoxia-Inducible Factors (HIF) are important transcriptional factors that regulate hypoxic cellular responses and maintain oxygen homeostasis [47]. HIF complexes lead to upregulation of VEGF expression, a key protein in neovascular AMD and the main target of the current treatment options. Experimental evidence suggests that DNA methylation may modulate both HIF1A expression, indirectly influencing the expression of angiogenic genes, and VEGF expression directly. DNA methylation inhibitors (DAC) have been suggested to normalize VEGF levels in RPE and retinal endothelial cells by downregulating VEGF [48]. Human methylome studies remain to be conducted in order to strengthen this indirect evidence.

### 3.2. Histone Modifications

Histone acetylation is a reversible epigenetic mechanism regulated mainly by histone acetyltransferases (HATs) and histone deacetylases (HDACs). The induced alterations modify chromatin accessibility and transcriptional activity, thus modulating gene expression. Different types of HDACs have been linked to transcriptional repression during aging processes and neurodegenerative diseases [49,50]. Therefore, they have been investigated as potential pharmaceutical targets for the treatment of various pathologies [51,52,53].

During the natural history of AMD, RPE atrophy precedes the loss of photoreceptor (PR) cells, which are dependent on RPE viability [54,55]. Notably, Dubey et al. examined aged retinal tissues from both animal models and humans and demonstrated a significant deficiency of essential histone components in the RPE cells [56]. They highlight that the hypoacetylation they observed may partially explain an epigenetic mechanism of the aging process and RPE senescence. In parallel, other experimental studies have shown that inhibiting HDACs with pharmacological agents offers protection to RPE cells from oxidative stress. Thus, it is indirectly suggested that under retinal stress conditions, histone acetylation homeostasis is altered [57,58].

HDACs have also been investigated for their antiangiogenic properties. Histone acetylation is implicated in the regulation of the VEGF and HIF1A genes [59]. These factors are of great importance in the development of choroidal neovascularization (CNV), a hallmark of neovascular/wet AMD. Various HDAC inhibitors have been used in experimental studies, demonstrating promising results in terms of reducing VEGF expression. AN7, an HDAC inhibitor, has been shown to reduce VEGF expression in retinal endothelial cells and enhance tight junctions in RPE cells in animal models, preventing oxidative injury that could lead to CNV formation [60]. Trichostatin A (TSA), a similar agent, was found to produce comparable outcomes by the downregulation of VEGF and HIF1A, while also upregulating the pigment epithelium-derived factor (PEDF) and inhibiting the epithelial–mesenchymal transition of RPE cells [61]. Moreover, HDAC inhibitors appear to regulate clusterin expression, a protein found in drusen between the RPE cell layer and Bruch’s membrane [62,63]. Clusterin is thought to counteract angiogenesis and inflammation, but its exact role in the pathophysiology of AMD has yet to be elucidated [64,65]. Overall, current evidence suggests a significant role for an acetylation/deacetylation imbalance in RPE aging, but an AMD-specific signature has not been established yet. Further human-based studies are needed to support this hypothesis and eventually offer new therapeutic options.

### 3.3. Non-Coding RNAs

MicroRNAs (miRNAs) are small, single-stranded, non-coding molecules whose length is usually 17–25 nucleotides. Unlike mRNA, they do not encode proteins, but their role consists mainly of silencing gene expression by degrading the corresponding mRNA [66]. They are essential parts of various cellular pathways such as cell differentiation, apoptosis, organogenesis, and angiogenesis. Notably, many miRNAs have been found to play a crucial role during retinal development, while they have also been implicated in many ocular pathologies, including retinal dystrophies [67], retinopathy of prematurity (ROP) [68], glaucoma [69], diabetic retinopathy (DR) [70], retinoblastoma [71], and ocular autoimmune diseases [72].

MiRNAs have been linked to the pathogenetic processes of AMD, including oxidative stress, inflammation, lipid metabolism, and neovascularization [73]. Upregulation of miR146a has been identified, induced by ROS and pro-inflammatory cytokines produced during oxidative stress in AMD. miRNA146a suppresses CFH, which represses inflammatory responses, as stated above [74]. Another significant miRNA implicated in oxidative stress is miR27a. miR27a has been characterized as an oxidative stress miRNA because its upregulation represses FOXO1 expression. The latter is part of the FOXO group, proteins regulating oxidative stress, autophagy, and apoptosis. Ren et al. found that RPE cell injury due to LED light exposure in mouse models was aggravated when miR27a was overexpressed or FOXO1 was downregulated [75]. Moreover, there are some miRNAs that appear to offer protective properties to RPE cells against oxidative stress-induced cell death. miR-1246 has an anti-apoptotic effect by reducing caspase-3 and caspase-14 levels, proteins involved in the apoptotic process [8]. MiR23a and miR274a have comparable properties, inhibiting the upregulation of Fas, an apoptotic factor activating the P53 pathways. Fas has been found to be overexpressed in AMD patients, leading to RPE apoptosis, while miR23a and miR274a were found at decreased levels in patients’ blood [76,77].

Drusen lesions in AMD consist mainly of lipid accumulations and lipoproteins. Dysregulated lipid metabolism, involving impaired cholesterol homeostasis, defective reverse cholesterol transport, and excessive lipid accumulations within Bruch’s membrane, is associated with AMD pathogenesis [78,79,80]. As post-transcriptional modulators of multiple genes, miRNAs are also involved in lipid metabolism pathways [79]. Among these, miR34a is one of the most studied lipid-associated miRNAs in AMD patients. miR34a induces the suppression of HNF4a under metabolic stress conditions, resulting in decreased expression of apolipoprotein B (ApoB) and microsomal triglyceride transfer protein (MTP), two significant regulators of lipoprotein assembly and secretion [79,81]. Notably, the dysregulation of this pathway is further supported by its significantly increased expression in macular regions of AMD patients [82]. Additional evidence implicates miR27b and miR223, which regulate cholesterol and triglyceride metabolism. Elevated circulating levels of them have been detected in AMD patients, and it is hypothesized that they are involved in lipid metabolic networks [83,84]. Furthermore, defective cholesterol efflux by the RPE is considered a major event in drusen formation, and it is mainly controlled through the reverse cholesterol transport pathway. miR-33, miR-128, and miR-148a negatively regulate ABCA1, a cholesterol transporter, resulting in reduced cholesterol efflux, while miR-223 appears to indirectly enhance ABCA1 expression and cholesterol removal [83,85,86]. Although this evidence originates from cardiovascular disease models, the association of miRNAs in the cholesterol pathway, a major contributor to AMD biogenesis, should be highlighted, and further research is needed to establish more robust conclusions.

Angiogenesis pathways are also modulated by the expression of some miRNAs. Most of them are implicated in VEGF-mediated processes. Inhibition of miR23 and miR27 was shown to enhance antiangiogenic protein expression both in vitro and in vivo [87]. miR205-5p targets VEGFA through the signaling pathway of phosphatidylinositol-3-kinase/protein kinase B (Akt) (PI3K)/PKB. It has been found to be downregulated in AMD patients, thus enhancing VEGFA expression [88]. Similar mechanisms of angiogenesis repression are regulated by miR93 and mi302d, which increase VEGFA secretion and inhibit the EMT process mediated by the transforming growth factor beta (TGF-β) [89]. Apart from VEGF-dependent pathways, there are also miRNAs that interfere with actin skeleton signaling, endothelial cell migration, and proliferation processes through modulation of proteins of Rho signaling downstream, as well as mTOR pathways [90,91].

#### 3.3.1. MiRNAs as Biomarkers and Therapeutic Targets

Circulating miRNAs are characterized by increased stability, rendering their detection in biological fluids easily performable, which prompted investigation of their potential as possible minimally invasive biomarkers for AMD diagnosis, phenotyping, and management [73,92]. Grassman et al. conducted one of the first large-scale studies identifying a distinct circulating miRNA profile in late-stage neovascular AMD patients [91]. This evidence supports the theory that peripheral miRNA signatures may discriminate affected individuals from controls. Similarly, distinct miRNA profiles between dry and wet AMD subjects have been detected by Szemraj et al. with miR-661 and miR-3121 being elevated in dry AMD, while miR-4258 and miR-889 were associated with wet AMD [93]. Importantly, miRNA profiles may differ among biological compartments examined, as identified by Menard et al., who detected different patterns of miRNA that only partially overlapped between vitreous humor and plasma in neovascular AMD subjects [94]. More recently, serum miR-29a-3p and miR-192-5p were also found to be increased in wet AMD patients compared to controls, further corroborating the miRNAs’ potential role as biomarkers [95].

Apart from diagnosis, miRNA profiling may also provide predictive information regarding treatment response. Yen et al., in their prospective study, identified elevated levels of miR-195-5p and miR-185-5p in the tears of anti-VEGF responders among treatment-naive wet AMD patients [96]. However, the clinical applicability of these biomarkers remains limited, as considerable heterogeneity exists across studies in method standardization (biological fluid examined, miRNA extraction, normalization methods, AMD phenotype) and miRNA signature reports [97].

The ability of miRNAs to modulate different components of angiogenic, inflammatory, and stress-response pathways in AMD has prompted investigators to examine the possibility of miRNA-based therapeutic agents [98]. Conceptually, the restoration of deficient miRNAs with synthetic mimics or inhibition of pathogenic miRNAs with antisense oligonucleotides or anti-miRs has been suggested as a potential therapeutic target [92]. For instance, in a preclinical setting of a laser-induced CNV mouse model, the restoration of miR93 expression reduced VEGFA expression and attenuated the choroidal neovascularization, indicating miRNA replacement can modulate pathogenic angiogenesis [99]. In parallel, manipulation of miR-126 has been shown to alter experimental CNV, further supporting the therapeutic potential of targeting angiogenesis-associated miRNAs [100]. Conversely, intravitreal inhibition of miR-142-3p using anti-miRs has been shown to suppress CNV and reduce choroidal neovascularization in vivo [101]. Despite the promising preclinical evidence, the pleiotropic effects of miRNAs and the inconsistent tissue–specific delivery, stability, and dose control remain major challenges [92,102]. While miRNAs seem attractive candidates for diagnosis, stratification, and targeted therapy, their clinical application will require further standardized studies and rigorous validation of ocular delivery strategies [97].

#### 3.3.2. Other Non-Coding RNAs

Besides miRNAs, long non-coding RNAs (lncRNAs) are also implicated in AMD pathophysiology. In a transcriptomic analysis of early-stage AMD tissue, researchers identified 64 differentially expressed lncRNAs, suggesting potential dysregulation of lncRNAs during the early stages of the disease [103]. MALAT1 has been extensively studied among the individual lncRNAs and has been linked to pathological angiogenesis. In an experimental CNV model, its expression was found to be increased in the RPE–complex, while MALAT1 knockout models were characterized by reduced vascular leakage, decreased CNV formation, endothelial proliferation, and migration. MALAT1 functions as an miR17-5p sponge, regulating VEGFA and ETS1 expression, which indicates the presence of a potential axis in choroidal angiogenesis [104]. Moreover, MEG3 has also been implicated in RPE homeostasis, as preclinical evidence suggests it protects RPE differentiation and limits mitochondrial oxidative stress through the miR-7-5p/PAX6 axis [105]. Overall, evidence linking lncRNAs to AMD pathogenesis remains experimental, and further human AMD studies are required to establish robust conclusions [106].

Circular RNAs (circRNAs) also represent a type of non-coding RNAs but have been less extensively studied in AMD. Liu et al. detected 100 differentially expressed circRNAs in RPE-choroid-sclera complexes in their laser-induced CNV mouse model, along with predictive networks implicating circRNAs, miRNAs, and mRNAs in immune, extracellular matrix, and inflammatory signaling pathways [107]. Similarly, in integrative analyses recently conducted, researchers have identified circRNAs regulatory networks associated with neovascular AMD; however, the evidence originates from bioinformatics or preclinical research. Therefore, circRNAs should be considered as emerging epigenetic regulators, requiring extensive research in human AMD tissue [108] (Table 1).

## 4. Gut Microbiota

### 4.1. Gut Microbiota Dysbiosis

Among ocular diseases, AMD is the most closely connected to the gut microbiota, as its dysbiosis can influence the progression of AMD through systemic inflammatory and metabolic pathways [109]. Individuals with gut dysbiosis may present chronic low-grade inflammation, oxidative stress, retinal pigment epithelium dysfunction, dysregulated complement activity, and pathological angiogenesis, all of which represent key mechanisms implicated in AMD pathogenesis [110]. The physiological gut microbiota comprises a diverse community of microorganisms that contribute to intestinal and systemic homeostasis. Under certain conditions, alterations in microbial composition, diversity, or function may disrupt this balance and promote dysbiosis [111]. Several studies have investigated whether such alterations are present in patients with AMD compared with unaffected individuals [112].

Current evidence suggests that there is no single, well-defined gut microbiome phenotype associated with AMD [113]. A significant role is played by environmental and ethnic differences between individuals that influence both genetic background and dietary habits [113]. Several studies have also suggested that gut microbial composition and abundance may vary according to disease severity. In particular, differences have been reported between patients with intermediate and advanced AMD, involving both microbial taxa and microbiota-derived metabolites [114]. Patients with advanced AMD showed an increased abundance of Desulfovibrionales and members of the class Clostridia compared with intermediate AMD cases and controls [114]. However, current human evidence remains limited, and most studies have compared patients with advanced or neovascular AMD with unaffected controls rather than directly evaluating consecutive stages of the disease [115].

Among the recent studies, several bacterial taxa have been reported to differ between patients with AMD and unaffected controls. In one of the first metagenomic studies, Zinkernagel et al. found that patients with neovascular AMD (nAMD) had an increased abundance of Anaerotruncus, Oscillibacter, Ruminococcus torques, and Eubacterium ventriosum, whereas Bacteroides eggerthii was more abundant in healthy controls [109]. Zysset-Burri et al. reported an enrichment of the class Negativicutes in patients with nAMD, while Oscillibacter and Bacteroides were more prevalent in controls, highlighting differences even among studies examining the same AMD phenotype [116]. Similarly, Zhang et al. observed a higher abundance of Proteobacteria, Bacteroidota, and Escherichia–Shigella in patients with AMD, whereas Firmicutes and the genera Blautia and Anaerostipes were more abundant in healthy controls [113]. Xue et al. also identified alterations in the gut microbiome of patients with AMD, including a decreased Firmicutes/Bacteroidetes ratio and enrichment of Ruminococcus callidus, Lactobacillus gasseri, and Prevotellaceae uSGB 2135 [113]. As previously mentioned, Parekh et al. identified significant differences in microbial composition between controls and patients with intermediate and advanced AMD, with several specific taxa differing between the two disease stages, while Dadgar et al. reported an increased abundance of Proteobacteria in patients with advanced AMD [115]. Taking into account the findings of these studies, current evidence suggests an association between the gut microbiome and AMD; however, the lack of consistency across studies indicates that a single, well-defined microbial signature for AMD has not yet been established.

### 4.2. Gut Microbiota–Derived Metabolites

#### 4.2.1. Short-Chain Fatty Acids and Retinal Homeostasis

SCFAs are among the most important metabolites produced by the gut microbiota through the degradation of complex dietary carbohydrates. The main SCFAs produced are Acetate, propionate, and Butyrate [117]. Several members of the gut microbiota contribute to SCFA production. Butyrate is primarily produced by bacteria such as Faecalibacterium, Roseburia, Eubacterium, and Clostridium [118,119]. Propionate is produced by Bacteroides, and Acetate is produced by a broader range of intestinal bacteria [120,121]. These microbial metabolites exert a wide range of physiological effects, including the maintenance of gut barrier integrity, regulation of the immune system, regulation of systemic and neuroinflammatory responses, epigenetic regulation (through HDAC inhibition), and involvement in several metabolic pathways [122].

Consequently, alterations in the abundance of SCFA-producing bacteria during gut dysbiosis may reduce SCFA availability [117,122]. As a result, this may compromise intestinal barrier integrity, leading to increased intestinal permeability, enhanced production of pro-inflammatory mediators, and activation of immune responses [121]. In animal models, a high-glycemic diet reduced fecal acetate and butyrate and aggravated AMD-like retinal degeneration, whereas switching to a low-glycemic diet restored SCFA levels and attenuated photoreceptor loss, suggesting a potential link between diet-induced microbial changes and retinal injury [123]. Direct supplementation studies further support a potential protective role for SCFAs in the retina. Exogenous butyrate administration has been shown to reduce retinal thinning and suppress microglial activation in experimental models of retinal degeneration [124], providing mechanistic evidence that SCFA availability can influence retinal homeostasis under specific experimental conditions. However, these findings are derived from preclinical models and do not establish a causal relationship between SCFA availability and AMD in humans. Reduced SCFA availability during dysbiosis may potentially contribute to retinal dysfunction through impaired gut barrier integrity and altered HDAC- and T-cell-mediated immune regulation, thereby promoting inflammatory pathways relevant to AMD pathogenesis.

#### 4.2.2. Lipopolysaccharide-Mediated Inflammation and Retinal Dysfunction

As mentioned, SCFAs may have a protective effect, in contrast to increased exposure to bacterial lipopolysaccharide (LPS), which may promote systemic inflammation [125,126]. LPS, also known as endotoxin, is a major component of the outer membrane of Gram-negative bacteria [127]. Gut dysbiosis may impair intestinal barrier integrity and increase intestinal permeability. Τhis may allow the translocation of LPS from the gut into systemic circulation, driving the development of systemic inflammation [128]. Specifically, circulating LPS can activate Toll-like receptor 4 (TLR4) in complex with MD2. This will contribute to the activation of NF-kB and the elevated production of pro-inflammatory cytokines such as IL-1β, IL-6, IL-8, and TNF-α [129]. LPS and the resulting chronic inflammatory state may disrupt retinal homeostasis through the activation of retinal microglia, macrophages, and vascular endothelial cells [130,131]. As a result, LPS-mediated systemic inflammation may affect the retinal pigment epithelium (RPE) and promote inflammatory and angiogenic responses relevant to AMD [132,133]. Experimental studies in P23H rats have shown that systemic exposure to LPS can exacerbate retinal inflammation and neurodegeneration [131]. Evidence from human studies also supports a potential association between LPS exposure and AMD. In a prospective population-based study, Larsen et al. found that higher plasma levels of esterified 3-hydroxy fatty acids, used as a proxy for LPS burden, were associated with an increased risk of incident early AMD [134]. However, no significant association was observed with incident advanced AMD, and a causal relationship between circulating LPS and AMD has not yet been established [135].

#### 4.2.3. Bile Acids, Tryptophan Metabolites, and Trimethylamine-N-Oxide (TMAO)

Bile acids represent another class of metabolites influenced by the gut microbiota. They are classified into primary and secondary bile acids. Primary bile acids are synthesized in the liver, and they may undergo modifications by the gut microbiota, resulting in the formation of secondary bile acids [136]. Alterations in gut microbiota may modify bile acid metabolism and potentially affect retinal homeostasis. Experimental studies have shown that tauroursodeoxycholic acid (TUDCA) reduces oxidative stress and protects photoreceptors from degeneration [134]. Additionally, taurocholic acid (TCA) preserves RPE integrity and inhibits VEGF-induced angiogenic responses in vitro [137]. Importantly, reduced levels of TUDCA and TCA have also been reported in patients with advanced AMD compared with intermediate AMD and controls, although their mechanistic role in AMD remains unclear [138].

Tryptophan-derived metabolites may represent another link between the microbiota and retinal homeostasis. Gut bacteria can convert tryptophan into bioactive indole derivatives, several of which modulate the aryl hydrocarbon receptor (AhR) [139]. AhR is expressed in the retina, choroid, and RPE and can influence inflammatory, oxidative, and angiogenic pathways relevant to AMD [139]. Human studies also indicate altered tryptophan metabolism in AMD. Increased 3-hydroxykynurenine and a reduced KYNA/3HK ratio have been reported in dry AMD, while increased kynurenine and reduced 5-HTP were observed in exudative AMD [140,141,142].

Lastly, Trimethylamine-N-oxide (TMAO) is described in the literature as a potential metabolite that may affect the retina. A recent metabolomic analysis reported markedly increased TMAO levels in the RPE/choroid of aged human donors [142]. However, this study associates increased TMAO levels more with aging than with AMD itself [142]. TMAO has been associated with oxidative stress, inflammation, and endothelial dysfunction. Experimental studies demonstrated that TMAO increases ROS production and activates the NLRP3 inflammasome, promoting inflammatory responses and endothelial dysfunction [143]. Moreover, TMAO can activate MAPK and NF-κB signaling in vascular endothelial cells, further promoting vascular inflammation [144]. Although these mechanisms could potentially affect retinal and choroidal homeostasis, the direct contribution of TMAO to AMD remains poorly established [142] (Table 2).

## 5. Microbiota–Epigenome Crosstalk in AMD

The gut microbiota and epigenetic regulation may represent interacting components of a broader network linking environmental exposures to retinal molecular responses in AMD. Epigenetic mechanisms are influenced by metabolic and inflammatory signals, while gut-derived metabolites may modulate these pathways. Emerging evidence suggests that microbiota-derived factors may contribute to AMD pathogenesis, in part through modulation of host gene expression.

Particularly, SCFAs have shown the most established mechanistic link between the microbiome and epigenetic regulation, since butyrate can inhibit HDACs, modulating chromatin accessibility and transcription, while SCFAs also influence immune responses and intestinal barrier integrity [122,124]. Altered SCFA availability has been associated with inflammatory, oxidative, vascular, and neurodegenerative processes in retinal diseases, including AMD [124]; reduced SCFA availability during dysbiosis may promote both systemic inflammation and epigenetic dysregulation relevant to retinal homeostasis. Moreover, recent Mendelian randomization analyses have linked specific gut microbial taxa with epigenetic aging phenotypes and advanced AMD outcomes [145,146]. In addition, microbiota-related gene networks converge on pathways involved in inflammation, lipid metabolism, VEGF signaling, and oxidative stress, and other microbial metabolites appear to influence additional epigenetically regulated pathways, including NF-κB, MAPK, PI3K–AKT, TNF, VEGF, Toll-like receptor, and NLRP3 signaling [21,147].

Overall, current evidence supports a coherent microbiota–metabolite–epigenome–retina axis, with SCFA-mediated HDAC regulation representing the most well-established mechanistic connection. Direct evidence for microbiota-driven epigenetic alterations in human AMD remains limited, with the need for integrated multi-omics studies to establish causality and translational relevance (Figure 1).

## 6. Limitations of Current Evidence and Future Directions

Existing evidence connecting epigenetic dysregulation and gut microbiota changes to AMD is limited by considerable variability across studies due to their study design, populations, disease stage, biological samples, and analytical methods. Much of what we know about epigenetic changes comes from animal or cell-based models, peripheral blood samples, or postmortem retinal tissue. Moreover, most studies are cross-sectional or preclinical, which makes it difficult to establish causality or determine how these molecular changes relate temporally to the onset and progression of the disease. Future studies should focus on prospective, longitudinal cohort studies that use clearly defined AMD phenotypes and consistent sampling protocols. Future therapeutic approaches targeting epigenetic pathways, non-coding RNAs, diet, or microbiota-derived metabolites are still at an early stage. Further clinical studies are therefore needed to determine whether targeting the microbiome–epigenome interaction can provide meaningful benefit in AMD prevention or as an adjunct to existing therapies.

## 7. Conclusions

AMD pathogenetic mechanisms seem to be even more complex, interacting with the established pathways. Epigenetic alterations and gut microbiota dysbiosis converge with AMD-induced oxidative stress, inflammation, complement activation, and angiogenesis. Moreover, microbiota-derived metabolites—including short-chain fatty acids, lipopolysaccharide, bile acids, and TMAO- share common inflammatory and oxidative pathogenetic pathways, which shows an integrated pathogenic network rather than isolated disease mechanisms. Further longitudinal and multi-omics studies are needed to clarify causality and determine whether these mechanisms can serve as reliable biomarkers or therapeutic targets in AMD.

## Figures and Tables

**Figure 1:**
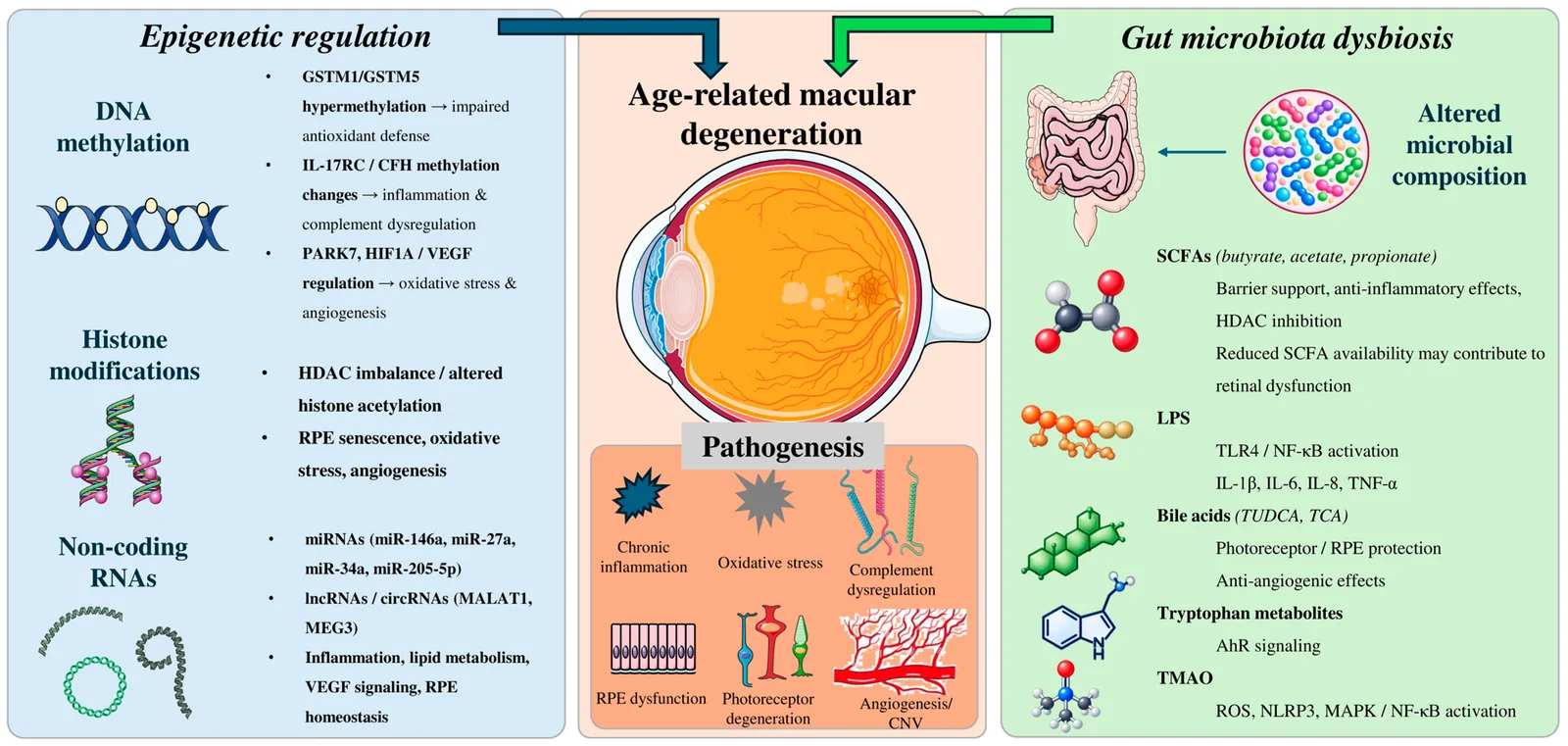
Proposed mechanisms linking epigenetic regulation and gut microbiota dysbiosis with age-related macular degeneration (AMD). Epigenetic alterations, including DNA methylation, histone modifications, and dysregulation of non-coding RNAs, may influence pathways involved in oxidative stress, inflammation, complement regulation, angiogenesis, and retinal pigment epithelium (RPE) homeostasis. In parallel, gut microbiota dysbiosis may alter microbial composition and the production of microbiota-derived metabolites, including short-chain fatty acids (SCFAs), lipopolysaccharide (LPS), bile acids, tryptophan metabolites, and trimethylamine-N-oxide (TMAO). These factors may affect intestinal barrier integrity, immune and inflammatory signaling, oxidative stress, and angiogenic pathways. The convergence of these epigenetic and microbiota-related mechanisms may contribute to key features of AMD pathogenesis, including chronic inflammation, oxidative stress, complement dysregulation, RPE dysfunction, photoreceptor degeneration, and choroidal neovascularization (CNV).

**Table 1:** Epigenetic regulations and their role in AMD.

Epigenetic Regulation	Target	Alterations in AMD	Pathogenesis	Study Type
DNA methylation [34,35,36]	GSTM1/GSTM5	Hypermethylation with reduced expression	Impaired antioxidant defense and increased oxidative stress	Human tissue study
DNA methylation [43,45]	IL-17RC, CFH	Altered promoter methylation	Inflammation and complement dysregulation	Human peripheral blood study; in vitro cybrid study
DNA methylation [27,37,38,48]	PARK7, HIF1A/VEGF	Epigenetic regulation of expression	Oxidative stress and angiogenesis	Human retinal study; animal model; in vitro human cell study
Histone modifications [56,57,58,59,60,61]	HDACs, histone acetylation	Altered acetylation balance in RPE	RPE senescence, oxidative stress, and angiogenesis	Human tissue; in vitro; animal models
MiRNAs [74,75,79,81,82,88]	miR-146a, miR-27a, miR-34a, miR-205-5p	Dysregulated expression	Inflammation, oxidative stress, lipid metabolism, and VEGF signaling	Human tissue; experimental/in vitro studies
Therapeutic miRNAs [99,100,101]	miR-93, miR-126, miR-142-3p	Experimental modulation alters CNV	Potential RNA-based therapeutic targets	Animal experimental CNV models
NcRNAs [103,104,105,106,107,108]	MALAT1, MEG3, circRNAs	Dysregulated mainly in preclinical models	Angiogenesis, RPE homeostasis, and inflammatory signaling	Human transcriptomic and blood studies; in vitro

AMD: age-related macular degeneration; CFH: complement factor H; circRNAs: circular RNAs; CNV: choroidal neovascularization; GSTM1/GSTM5: glutathione S-transferase mu 1/5; HDACs: histone deacetylases; HIF1A: hypoxia-inducible factor 1 alpha; IL-17RC: interleukin-17 receptor C; MALAT1: metastasis-associated lung adenocarcinoma transcript 1; MEG3: maternally expressed gene 3; miRNAs: microRNAs; NcRNAs: non-coding RNAs; PARK7: Parkinsonism-associated deglycase 7; RPE: retinal pigment epithelium; VEGF: vascular endothelial growth factor.

**Table 2:** Gut microbiota-derived metabolites and their retinal effects in AMD.

Metabolites	Compounds	Effect on Retina/RPE	Evidence in AMD Patients	Study Type
SCFAs [117,121,122,123,124]	Butyrate, Acetate, Propionate	Anti-inflammatory; barrier support; HDAC inhibition	↓ levels in advanced AMD	Animal/preclinical studies
LPS [129,130,131,132,133,135]	Endotoxin	Pro-inflammatory (TLR4/NF-κB activation)	↑ LPS-proxy associated with incident early AMD	Human prospective study; animal models; in vitro studies
Bile acids [134,137,138]	TUDCA, TCA	Photoreceptor/RPE protection; anti-angiogenic	↓ levels in advanced AMD	Preclinical experimental study; in vitro study
Tryptophan metabolites [139,140,141,142]	Kynurenine, 3-HK, 5-HTP	AhR-mediated inflammatory/oxidative modulation	Altered ratios in dry vs. wet AMD	Human clinical and tissue studies
TMAO [142,143,144]	-	Pro-inflammatory (NLRP3, MAPK/NF-κB)	↑ in aged RPE/choroid (aging-associated)	Human tissue; in vitro; experimental mechanistic evidence

AhR: Aryl hydrocarbon receptor; AMD: Age-related macular degeneration; 3-HK: 3-Hydroxykynurenine; 5-HTP: 5-Hydroxytryptophan; LPS: Lipopolysaccharide; MAPK: Mitogen-activated protein kinase; NF-κB: Nuclear factor kappa-light-chain-enhancer of activated B cells; NLRP3: NOD-, LRR-, and pyrin domain-containing protein 3; RPE: Retinal pigment epithelium; SCFAs: Short-chain fatty acids; TCA: Taurocholic acid; TLR4: Toll-like receptor 4; TMAO: Trimethylamine-N-oxide; TUDCA: Tauroursodeoxycholic acid.

## Data Availability

No new data were created or analyzed in this study. Data sharing is not applicable to this article.

## References

[1] Mitchell P., Liew G., Gopinath B., Wong T.Y. (2018). Age-related macular degeneration. Lancet.

[2] Al-Zamil W.M., Yassin S.A. (2017). Recent developments in age-related macular degeneration: A review. Clin. Interv. Aging.

[3] Tang S., Yang J., Xiao B., Wang Y., Lei Y., Lai D., Qiu Q. (2024). Aberrant Lipid Metabolism and Complement Activation in Age-Related Macular Degeneration. Investig. Ophthalmol. Vis. Sci..

[4] Buonfiglio F., Korb C.A., Stoffelns B., Pfeiffer N., Gericke A. (2024). Recent Advances in Our Understanding of Age-Related Macular Degeneration: Mitochondrial Dysfunction, Redox Signaling, and the Complement System. Aging Dis..

[5] Jiang F., Ma J., Lei C., Zhang Y., Zhang M. (2025). Age-Related Macular Degeneration: Cellular and Molecular Signaling Mechanisms. Int. J. Mol. Sci..

[6] Chen K.Y., Chan H.C., Lin W.W., Chan C.M. (2026). Mitochondrial dynamics and their role in the pathogenesis of age-related macular degeneration: A comprehensive review. Redox Biol..

[7] Chu L., Bi C., Wang C., Zhou H. (2024). The Relationship between Complements and Age-Related Macular Degeneration and Its Pathogenesis. J. Ophthalmol..

[8] Wilke G.A., Apte R.S. (2024). Complement regulation in the eye: Implications for age-related macular degeneration. J. Clin. Investig..

[9] Huang L., Ye L., Li R., Zhang S., Qu C., Li S., Li J., Yang M., Wu B., Chen R. (2023). Dynamic human retinal pigment epithelium (RPE) and choroid architecture based on single-cell transcriptomic landscape analysis. Genes Dis..

[10] Tan Y., Huang J., Li D., Zou C., Liu D., Qin B. (2023). Single-cell RNA sequencing in dissecting microenvironment of age-related macular degeneration: Challenges and perspectives. Ageing Res. Rev..

[11] Lin J.B., Santeford A., Colasanti J.J., Lee Y., Shah A.V., Wang T.J., Ruzycki P.A., Apte R.S. (2024). Targeting cell-type-specific, choroid-peripheral immune signaling to treat age-related macular degeneration. Cell Rep. Med..

[12] Voigt A.P., Mullin N.K., Mulfaul K., Lozano L.P., Navratil E.M., Flamme-Wiese M.J., Lavine J.A., Fingert J.H., Tucker B.A., Stone E.M. (2026). Single-Cell Gene Expression and eQTL Analyses in the Human Retina, RPE, and Choroid in Macular Degeneration. bioRxiv.

[13] Wang N., Wang Y., Zhang L., Yang W., Fu S. (2025). Molecular Mechanisms of Epithelial-Mesenchymal Transition in Retinal Pigment Epithelial Cells: Implications for Age-Related Macular Degeneration (AMD) Progression. Biomolecules.

[14] Mazzio E.A., Soliman K.F. (2012). Basic concepts of epigenetics: Impact of environmental signals on gene expression. Epigenetics.

[15] Gibney E.R., Nolan C.M. (2010). Epigenetics and gene expression. Heredity.

[16] Portela A., Esteller M. (2010). Epigenetic modifications and human disease. Nat. Biotechnol..

[17] He S., Li X., Chan N., Hinton D.R. (2013). Review: Epigenetic mechanisms in ocular disease. Mol. Vis..

[18] Borrelli E., Neri G., Berni A., Olivieri C., Chhablani J., Corradetti G., Dolz-Marco R., Reiter G.S., Zur D., Bandello F. (2026). Review of emerging imaging findings to reveal a broader spectrum of lesions in AMD. Graefes Arch. Clin. Exp. Ophthalmol..

[19] Hjelmeland L.M. (2011). Dark matters in AMD genetics: Epigenetics and stochasticity. Investig. Ophthalmol. Vis. Sci..

[20] Liu M.M., Chan C.C., Tuo J. (2012). Genetic mechanisms and age-related macular degeneration: Common variants, rare variants, copy number variations, epigenetics, and mitochondrial genetics. Hum. Genom..

[21] Kisswani D., Carroll C., Valdes-Mora F., Rutar M. (2025). Epigenetic Alterations in Age-Related Macular Degeneration: Mechanisms and Implications. Int. J. Mol. Sci..

[22] Oblak L., van der Zaag J., Higgins-Chen A.T., Levine M.E., Boks M.P. (2021). A systematic review of biological, social and environmental factors associated with epigenetic clock acceleration. Ageing Res. Rev..

[23] Fleckenstein M., Schmitz-Valckenberg S., Chakravarthy U. (2024). Age-Related Macular Degeneration: A Review. JAMA.

[24] Sobrin L., Seddon J.M. (2014). Nature and nurture- genes and environment- predict onset and progression of macular degeneration. Prog. Retin. Eye Res..

[25] Moore L.D., Le T., Fan G. (2013). DNA methylation and its basic function. Neuropsychopharmacology.

[26] Xu C., Fu X., Qin H., Yao K. (2024). Traversing the epigenetic landscape: DNA methylation from retina to brain in development and disease. Front. Cell. Neurosci..

[27] Advani J., Mehta P.A., Hamel A.R., Mehrotra S., Kiel C., Strunz T., Corso-Diaz X., Kwicklis M., van Asten F., Ratnapriya R. (2024). QTL mapping of human retina DNA methylation identifies 87 gene-epigenome interactions in age-related macular degeneration. Nat. Commun..

[28] Ghannai S., Ribeiro E., Pereira B., Brito M., Camacho P. (2026). The potential of DNA methylation as a biomarker for age-related macular degeneration: A systematic review. Arch.Soc. Esp. Oftalmol. (Engl. Ed.).

[29] Ozawa Y. (2020). Oxidative stress in the light-exposed retina and its implication in age-related macular degeneration. Redox Biol..

[30] Domènech E.B., Marfany G. (2020). The Relevance of Oxidative Stress in the Pathogenesis and Therapy of Retinal Dystrophies. Antioxidants.

[31] Kawashima H., Ozawa Y., Toda E., Homma K., Osada H., Narimatsu T., Nagai N., Tsubota K. (2020). Neuroprotective and vision-protective effect of preserving ATP levels by AMPK activator. FASEB J..

[32] Narimatsu T., Ozawa Y., Miyake S., Kubota S., Hirasawa M., Nagai N., Shimmura S., Tsubota K. (2013). Disruption of cell-cell junctions and induction of pathological cytokines in the retinal pigment epithelium of light-exposed mice. Investig. Ophthalmol. Vis. Sci..

[33] Rozing M.P., Durhuus J.A., Krogh Nielsen M., Subhi Y., Kirkwood T.B., Westendorp R.G., Sorensen T.L. (2020). Age-related macular degeneration: A two-level model hypothesis. Prog. Retin. Eye Res..

[34] Hunter A., Spechler P.A., Cwanger A., Song Y., Zhang Z., Ying G.S., Hunter A.K., Dezoeten E., Dunaief J.L. (2012). DNA methylation is associated with altered gene expression in AMD. Investig. Ophthalmol. Vis. Sci..

[35] Coles B.F., Kadlubar F.F. (2003). Detoxification of electrophilic compounds by glutathione S-transferase catalysis: Determinants of individual response to chemical carcinogens and chemotherapeutic drugs?. Biofactors.

[36] Singhal S.S., Singh S.P., Singhal P., Horne D., Singhal J., Awasthi S. (2015). Antioxidant role of glutathione S-transferases: 4-Hydroxynonenal, a key molecule in stress-mediated signaling. Toxicol. Appl. Pharmacol..

[37] Upadhyay M., Milliner C., Bell B.A., Bonilha V.L. (2020). Oxidative stress in the retina and retinal pigment epithelium (RPE): Role of aging, and DJ-1. Redox Biol..

[38] Bonilha V.L., Bell B.A., Rayborn M.E., Yang X., Kaul C., Grossman G.H., Samuels I.S., Hollyfield J.G., Xie C., Cai H. (2015). Loss of DJ-1 elicits retinal abnormalities, visual dysfunction, and increased oxidative stress in mice. Exp. Eye Res..

[39] Kawa M.P., Machalinska A., Roginska D., Machalinski B. (2014). Complement system in pathogenesis of AMD: Dual player in degeneration and protection of retinal tissue. J. Immunol. Res..

[40] Romero-Vazquez S., Llorens V., Soler-Boronat A., Figueras-Roca M., Adan A., Molins B. (2021). Interlink between Inflammation and Oxidative Stress in Age-Related Macular Degeneration: Role of Complement Factor H. Biomedicines.

[41] Ardeljan D., Wang Y., Park S., Shen D., Chu X.K., Yu C.R., Abu-Asab M., Tuo J., Eberhart C.G., Olsen T.W. (2014). Interleukin-17 retinotoxicity is prevented by gene transfer of a soluble interleukin-17 receptor acting as a cytokine blocker: Implications for age-related macular degeneration. PLoS ONE.

[42] Nassar K., Grisanti S., Elfar E., Luke J., Luke M., Grisanti S. (2015). Serum cytokines as biomarkers for age-related macular degeneration. Graefes Arch. Clin. Exp. Ophthalmol..

[43] Wei L., Liu B., Tuo J., Shen D., Chen P., Li Z., Liu X., Ni J., Dagur P., Sen H.N. (2012). Hypomethylation of the IL17RC promoter associates with age-related macular degeneration. Cell Rep..

[44] Liang G., Ma W., Luo Y., Yin J., Hao L., Zhong J. (2022). Identification of differentially expressed and methylated genes and construction of a co-expression network in age-related macular degeneration. Ann. Transl. Med..

[45] Nashine S., Chwa M., Kazemian M., Thaker K., Lu S., Nesburn A., Kuppermann B.D., Kenney M.C. (2016). Differential Expression of Complement Markers in Normal and AMD Transmitochondrial Cybrids. PLoS ONE.

[46] Fleckenstein M., Keenan T.D.L., Guymer R.H., Chakravarthy U., Schmitz-Valckenberg S., Klaver C.C., Wong W.T., Chew E.Y. (2021). Age-related macular degeneration. Nat. Rev. Dis. Primers.

[47] Henning Y., Blind U.S., Larafa S., Matschke J., Fandrey J. (2022). Hypoxia aggravates ferroptosis in RPE cells by promoting the Fenton reaction. Cell Death Dis..

[48] Xie M., Tian J., Luo Y., Wei L., Lin S., Tang S. (2014). Effects of 5-aza-2′-deoxycytidine and trichostatin A on high glucose- and interleukin-1beta-induced secretory mediators from human retinal endothelial cells and retinal pigment epithelial cells. Mol. Vis..

[49] Morris M.J., Monteggia L.M. (2013). Unique functional roles for class I and class II histone deacetylases in central nervous system development and function. Int. J. Dev. Neurosci..

[50] Razick D.I., Akhtar M., Wen J., Alam M., Dean N., Karabala M., Ansari U., Ansari Z., Tabaie E., Siddiqui S. (2023). The Role of Sirtuin 1 (SIRT1) in Neurodegeneration. Cureus.

[51] Pedro Ferreira J., Pitt B., Zannad F. (2021). Histone deacetylase inhibitors for cardiovascular conditions and healthy longevity. Lancet Healthy Longev..

[52] Cantley M.D., Haynes D.R. (2013). Epigenetic regulation of inflammation: Progressing from broad acting histone deacetylase (HDAC) inhibitors to targeting specific HDACs. Inflammopharmacology.

[53] Glauben R., Sonnenberg E., Zeitz M., Siegmund B. (2009). HDAC inhibitors in models of inflammation-related tumorigenesis. Cancer Lett..

[54] Chen L., Messinger J.D., Zhang Y., Spaide R.F., Freund K.B., Curcio C.A. (2020). SUBRETINAL DRUSENOID DEPOSIT IN AGE-RELATED MACULAR DEGENERATION: Histologic Insights Into Initiation, Progression to Atrophy, and Imaging. Retina.

[55] Christenbury J.G., Folgar F.A., O’Connell R.V., Chiu S.J., Farsiu S., Toth C.A., Age-related Eye Disease Study 2 Ancillary Spectral Domain Optical Coherence Tomography Study Group (2013). Progression of intermediate age-related macular degeneration with proliferation and inner retinal migration of hyperreflective foci. Ophthalmology.

[56] Dubey S.K., Dubey R., Prajapati S.C., Jung K., Mohan K., Liu X., Roney J., Tian W., Abney J., Giarmarco M.M. (2024). Histone deficiency and hypoacetylation in the aging retinal pigment epithelium. Aging Cell.

[57] Lugassy Y., Berent E., Tarony L., Jeries S., Ziv T., Savion N., Eldar-Finkelman H. (2025). HDAC inhibition protects RPE cells from oxidative stress via enhanced mitochondrial fusion, cytoskeletal repair, and Nrf-2 activation. Free Radic. Biol. Med..

[58] Berner A.K., Kleinman M.E. (2016). Therapeutic Approaches to Histone Reprogramming in Retinal Degeneration. Retinal Degenerative Diseases.

[59] Shiva Shankar T.V., Willems L. (2014). Epigenetic modulators mitigate angiogenesis through a complex transcriptomic network. Vasc. Pharmacol..

[60] Dahbash M., Sella R., Megiddo-Barnir E., Nisgav Y., Tarasenko N., Weinberger D., Rephaeli A., Livnat T. (2019). The Histone Deacetylase Inhibitor AN7, Attenuates Choroidal Neovascularization in a Mouse Model. Int. J. Mol. Sci..

[61] Chan N., He S., Spee C.K., Ishikawa K., Hinton D.R. (2015). Attenuation of choroidal neovascularization by histone deacetylase inhibitor. PLoS ONE.

[62] Suuronen T., Nuutinen T., Ryhanen T., Kaarniranta K., Salminen A. (2007). Epigenetic regulation of clusterin/apolipoprotein J expression in retinal pigment epithelial cells. Biochem. Biophys. Res. Commun..

[63] Gottlicher M., Minucci S., Zhu P., Kramer O.H., Schimpf A., Giavara S., Sleeman J.P., Lo Coco F., Nervi C., Pelicci P.G. (2001). Valproic acid defines a novel class of HDAC inhibitors inducing differentiation of transformed cells. EMBO J..

[64] Crabb J.W., Miyagi M., Gu X., Shadrach K., West K.A., Sakaguchi H., Kamei M., Hasan A., Yan L., Rayborn M.E. (2002). Drusen proteome analysis: An approach to the etiology of age-related macular degeneration. Proc. Natl. Acad. Sci. USA.

[65] Kim J.H., Kim J.H., Jun H.O., Yu Y.S., Min B.H., Park K.H., Kim K.W. (2010). Protective effect of clusterin from oxidative stress-induced apoptosis in human retinal pigment epithelial cells. Investig. Ophthalmol. Vis. Sci..

[66] Gemayel M.C., Bhatwadekar A.D., Ciulla T. (2021). RNA therapeutics for retinal diseases. Expert Opin. Biol. Ther..

[67] Conte I., Hadfield K.D., Barbato S., Carrella S., Pizzo M., Bhat R.S., Carissimo A., Karali M., Porter L.F., Urquhart J. (2015). MiR-204 is responsible for inherited retinal dystrophy associated with ocular coloboma. Proc. Natl. Acad. Sci. USA.

[68] Kim H., Kim J., Ryu J. (2022). Noncoding RNAs as a novel approach to target retinopathy of prematurity. Front. Pharmacol..

[69] Moazzeni H., Khani M., Elahi E. (2020). Insights into the regulatory molecules involved in glaucoma pathogenesis. Am. J. Med. Genet. C Semin. Med. Genet..

[70] Lampsas S., Agapitou C., Chatzirallis A., Papavasileiou G., Poulakis D., Pegka S., Theodossiadis P., Lambadiari V., Chatziralli I. (2025). MicroRNA (miRNA) in the Pathogenesis of Diabetic Retinopathy: A Narrative Review. Genes.

[71] Lande K., Gupta J., Ranjan R., Kiran M., Torres Solis L.F., Solis Herrera A., Aliev G., Karnati R. (2020). Exosomes: Insights from Retinoblastoma and Other Eye Cancers. Int. J. Mol. Sci..

[72] Wei Y., Li N., Zhao L., Yang C., Ma B., Li X., Wei R., Nian H. (2020). MicroRNAs and Autoimmune-Mediated Eye Diseases. Front. Cell Dev. Biol..

[73] Urbanska K., Stepien P.W., Nowakowska K.N., Stefaniak M., Osial N., Choragiewicz T., Toro M.D., Nowomiejska K., Rejdak R. (2022). The Role of Dysregulated miRNAs in the Pathogenesis, Diagnosis and Treatment of Age-Related Macular Degeneration. Int. J. Mol. Sci..

[74] Alexandrov P.N., Dua P., Lukiw W.J. (2014). Up-Regulation of miRNA-146a in Progressive, Age-Related Inflammatory Neurodegenerative Disorders of the Human CNS. Front. Neurol..

[75] Ren C., Hu W., Wei Q., Cai W., Jin H., Yu D., Liu C., Shen T., Zhu M., Liang X. (2021). MicroRNA-27a Promotes Oxidative-Induced RPE Cell Death through Targeting FOXO1. Biomed Res. Int..

[76] Lin H., Qian J., Castillo A.C., Long B., Keyes K.T., Chen G., Ye Y. (2011). Effect of miR-23 on oxidant-induced injury in human retinal pigment epithelial cells. Investig. Ophthalmol. Vis. Sci..

[77] Tasharrofi N., Kouhkan F., Soleimani M., Soheili Z.S., Kabiri M., Mahmoudi Saber M., Dorkoosh F.A. (2017). Survival Improvement in Human Retinal Pigment Epithelial Cells via Fas Receptor Targeting by miR-374a. J. Cell. Biochem..

[78] Conley Y.P., Thalamuthu A., Jakobsdottir J., Weeks D.E., Mah T., Ferrell R.E., Gorin M.B. (2005). Candidate gene analysis suggests a role for fatty acid biosynthesis and regulation of the complement system in the etiology of age-related maculopathy. Hum. Mol. Genet..

[79] Jun S., Datta S., Wang L., Pegany R., Cano M., Handa J.T. (2019). The impact of lipids, lipid oxidation, and inflammation on AMD, and the potential role of miRNAs on lipid metabolism in the RPE. Exp. Eye Res..

[80] Curcio C.A., Johnson M., Rudolf M., Huang J.D. (2011). The oil spill in ageing Bruch membrane. Br. J. Ophthalmol..

[81] Xu Y., Zalzala M., Xu J., Li Y., Yin L., Zhang Y. (2015). A metabolic stress-inducible miR-34a-HNF4alpha pathway regulates lipid and lipoprotein metabolism. Nat. Commun..

[82] Bhattacharjee S., Zhao Y., Dua P., Rogaev E.I., Lukiw W.J. (2016). microRNA-34a-Mediated Down-Regulation of the Microglial-Enriched Triggering Receptor and Phagocytosis-Sensor TREM2 in Age-Related Macular Degeneration. PLoS ONE.

[83] Vickers K.C., Sethupathy P., Baran-Gale J., Remaley A.T. (2013). Complexity of microRNA function and the role of isomiRs in lipid homeostasis. J. Lipid Res..

[84] Ertekin S., Yildirim O., Dinc E., Ayaz L., Fidanci S.B., Tamer L. (2014). Evaluation of circulating miRNAs in wet age-related macular degeneration. Mol. Vis..

[85] Marquart T.J., Allen R.M., Ory D.S., Baldan A. (2010). miR-33 links SREBP-2 induction to repression of sterol transporters. Proc. Natl. Acad. Sci. USA.

[86] Goedeke L., Fernandez-Hernando C. (2014). MicroRNAs: A connection between cholesterol metabolism and neurodegeneration. Neurobiol. Dis..

[87] Zhou Q., Gallagher R., Ufret-Vincenty R., Li X., Olson E.N., Wang S. (2011). Regulation of angiogenesis and choroidal neovascularization by members of microRNA-23~27~24 clusters. Proc. Natl. Acad. Sci. USA.

[88] Oltra M., Vidal-Gil L., Maisto R., Sancho-Pelluz J., Barcia J.M. (2020). Oxidative stress-induced angiogenesis is mediated by miR-205-5p. J. Cell. Mol. Med..

[89] Fuchs H.R., Meister R., Lotke R., Framme C. (2020). The microRNAs miR-302d and miR-93 inhibit TGFB-mediated EMT and VEGFA secretion from ARPE-19 cells. Exp. Eye Res..

[90] Zhou Q., Anderson C., Zhang H., Li X., Inglis F., Jayagopal A., Wang S. (2014). Repression of choroidal neovascularization through actin cytoskeleton pathways by microRNA-24. Mol. Ther..

[91] Grassmann F., Schoenberger P.G., Brandl C., Schick T., Hasler D., Meister G., Fleckenstein M., Lindner M., Helbig H., Fauser S. (2014). A circulating microrna profile is associated with late-stage neovascular age-related macular degeneration. PLoS ONE.

[92] Natoli R., Fernando N. (2018). MicroRNA as Therapeutics for Age-Related Macular Degeneration. Retinal Degenerative Diseases.

[93] Szemraj M., Bielecka-Kowalska A., Oszajca K., Krajewska M., Gos R., Jurowski P., Kowalski M., Szemraj J. (2015). Serum MicroRNAs as Potential Biomarkers of AMD. Med. Sci. Monit..

[94] Menard C., Rezende F.A., Miloudi K., Wilson A., Tetreault N., Hardy P., SanGiovanni J.P., De Guire V., Sapieha P. (2016). MicroRNA signatures in vitreous humour and plasma of patients with exudative AMD. Oncotarget.

[95] Dahmcke M., Busch M., Pfeil J.M., Brauckmann T., Schulz D., Omran W., Morawiec-Kisiel E., Wahler F., Paul S., Tayar A. (2023). Circulating MicroRNAs as Biomarker for Vessel-Associated Retinal Diseases. Ophthalmologica.

[96] Yen C.Y., Chiu C.M., Fang I.M. (2024). MicroRNA expression profiling in tears and blood as predictive biomarkers for anti-VEGF treatment response in wet age-related macular degeneration. Graefes Arch. Clin. Exp. Ophthalmol..

[97] Chen K.Y., Chan H.C., Chan C.M. (2026). Can MicroRNAs serve as reliable biomarkers and novel therapeutic targets in age-related macular degeneration? A systematic review and meta-analysis. J. Transl. Med..

[98] Wang S., Koster K.M., He Y., Zhou Q. (2012). miRNAs as potential therapeutic targets for age-related macular degeneration. Future Med. Chem..

[99] Wang L., Lee A.Y., Wigg J.P., Peshavariya H., Liu P., Zhang H. (2016). miRNA involvement in angiogenesis in age-related macular degeneration. J. Physiol. Biochem..

[100] Wang L., Lee A.Y., Wigg J.P., Peshavariya H., Liu P., Zhang H. (2016). miR-126 Regulation of Angiogenesis in Age-Related Macular Degeneration in CNV Mouse Model. Int. J. Mol. Sci..

[101] Roblain Q., Louis T., Yip C., Baudin L., Struman I., Caolo V., Lambert V., Lecomte J., Noel A., Heymans S. (2021). Intravitreal injection of anti-miRs against miR-142-3p reduces angiogenesis and microglia activation in a mouse model of laser-induced choroidal neovascularization. Aging.

[102] Martinez B., Peplow P.V. (2021). MicroRNAs in laser-induced choroidal neovascularization in mice and rats: Their expression and potential therapeutic targets. Neural Regen. Res..

[103] Zhu W., Meng Y.F., Xing Q., Tao J.J., Lu J., Wu Y. (2017). Identification of lncRNAs involved in biological regulation in early age-related macular degeneration. Int. J. Nanomed..

[104] Zhang X., Du S., Yang D., Jin X., Zhang Y., Wang D., Wang H., Zhang Y., Zhu M. (2023). LncRNA MALAT1 knockdown inhibits the development of choroidal neovascularization. Heliyon.

[105] Sun H.J., Zhang F.F., Xiao Q., Xu J., Zhu L.J. (2021). lncRNA MEG3, Acting as a ceRNA, Modulates RPE Differentiation Through the miR-7-5p/Pax6 Axis. Biochem. Genet..

[106] Qin L., Gao X., Lu X., Liu W., Tian J., Yuan G. (2025). Investigation of LncRNA Expression Profiles and Analysis of Immune-Related lncRNA-miRNA-mRNA Networks in Neovascular Age-Related Macular Degeneration. Comb. Chem. High Throughput Screen..

[107] Liu X., Zhang L., Wang J.H., Zeng H., Zou J., Tan W., Zhao H., He Y., Shi J., Yoshida S. (2020). Investigation of circRNA Expression Profiles and Analysis of circRNA-miRNA-mRNA Networks in an Animal (Mouse) Model of Age-Related Macular Degeneration. Curr. Eye Res..

[108] Zhang M., Wu J., Wang Y., Wan X., Liu H., Sun X. (2023). Identification of Cuproptosis-Related circRNA-miRNA-mRNA Network in Laser-Induced Choroidal Neovascularization Models and in Peripheral Blood Mononuclear Cells of Patients with Neovascular Age-Related Macular Degeneration. Ophthalmic Res..

[109] Zinkernagel M.S., Zysset-Burri D.C., Keller I., Berger L.E., Leichtle A.B., Largiader C.R., Fiedler G.M., Wolf S. (2017). Association of the Intestinal Microbiome with the Development of Neovascular Age-Related Macular Degeneration. Sci. Rep..

[110] Andriessen E.M., Wilson A.M., Mawambo G., Dejda A., Miloudi K., Sennlaub F., Sapieha P. (2016). Gut microbiota influences pathological angiogenesis in obesity-driven choroidal neovascularization. EMBO Mol. Med..

[111] Kho Z.Y., Lal S.K. (2018). The Human Gut Microbiome—A Potential Controller of Wellness and Disease. Front. Microbiol..

[112] Zhang Y., Wang T., Wan Z., Bai J., Xue Y., Dai R., Wang M., Peng Q. (2023). Alterations of the intestinal microbiota in age-related macular degeneration. Front. Microbiol..

[113] Xue W., Peng P., Wen X., Meng H., Qin Y., Deng T., Guo S., Chen T., Li X., Liang J. (2023). Metagenomic Sequencing Analysis Identifies Cross-Cohort Gut Microbial Signatures Associated With Age-Related Macular Degeneration. Investig. Ophthalmol. Vis. Sci..

[114] Parekh Z., Xiao J., Mani A., Evans Q., Phung C., Barba H.A., Xie B., Sidebottom A.M., Sundararajan A., Lin H. (2025). Fecal Microbial Profiles and Short-Chain Fatty Acid/Bile Acid Metabolomics in Patients With Age-Related Macular Degeneration: A Pilot Study. Investig. Ophthalmol. Vis. Sci..

[115] Dadgar N., Fung K., McClintic S., Metea C., Llorenc V., Saleh M., Nakamura Y.K., Jahrig C., Kiang L., Janowitz C. (2026). Age-related Eye Disease Studies Supplements and Genetic Risk Score Are Crucial Determinants of Intestinal Microbial Alterations in Advanced Age-Related Macular Degeneration. Ophthalmol. Sci..

[116] Zysset-Burri D.C., Morandi S., Herzog E.L., Berger L.E., Zinkernagel M.S. (2023). The role of the gut microbiome in eye diseases. Prog. Retin. Eye Res..

[117] Martin-Gallausiaux C., Marinelli L., Blottiere H.M., Larraufie P., Lapaque N. (2021). SCFA: Mechanisms and functional importance in the gut. Proc. Nutr. Soc..

[118] Martin-Gallausiaux C., Larraufie P., Jarry A., Beguet-Crespel F., Marinelli L., Ledue F., Reimann F., Blottiere H.M., Lapaque N. (2018). Butyrate Produced by Commensal Bacteria Down-Regulates Indolamine 2,3-Dioxygenase 1 (IDO-1) Expression via a Dual Mechanism in Human Intestinal Epithelial Cells. Front. Immunol..

[119] Snodgrass J.L., Velayudhan B.T. (2026). Butyrate-Producing Bacteria as a Keystone Species of the Gut Microbiome: A Systemic Review of Dietary Impact on Gut-Brain and Host Health. Int. J. Mol. Sci..

[120] Reichardt N., Duncan S.H., Young P., Belenguer A., McWilliam Leitch C., Scott K.P., Flint H.J., Louis P. (2014). Phylogenetic distribution of three pathways for propionate production within the human gut microbiota. ISME J..

[121] Falony G., Vlachou A., Verbrugghe K., De Vuyst L. (2006). Cross-feeding between Bifidobacterium longum BB536 and acetate-converting, butyrate-producing colon bacteria during growth on oligofructose. Appl. Environ. Microbiol..

[122] Fusco W., Lorenzo M.B., Cintoni M., Porcari S., Rinninella E., Kaitsas F., Lener E., Mele M.C., Gasbarrini A., Collado M.C. (2023). Short-Chain Fatty-Acid-Producing Bacteria: Key Components of the Human Gut Microbiota. Nutrients.

[123] Rowan S., Taylor A. (2018). Gut microbiota modify risk for dietary glycemia-induced age-related macular degeneration. Gut Microbes.

[124] Ciurariu E., Tirziu A.T., Varga N.I., Hirtie B., Alexandru A., Ivan C.S., Nicolescu L. (2025). Short-Chain Fatty Acids and the Gut-Retina Connection: A Systematic Review. Int. J. Mol. Sci..

[125] Mann E.R., Lam Y.K., Uhlig H.H. (2024). Short-chain fatty acids: Linking diet, the microbiome and immunity. Nat. Rev. Immunol..

[126] Violi F., Cammisotto V., Bartimoccia S., Pignatelli P., Carnevale R., Nocella C. (2023). Gut-derived low-grade endotoxaemia, atherothrombosis and cardiovascular disease. Nat. Rev. Cardiol..

[127] Kanjilal-Kolar S., Basu S.S., Kanipes M.I., Guan Z., Garrett T.A., Raetz C.R. (2006). Expression cloning of three Rhizobium leguminosarum lipopolysaccharide core galacturonosyltransferases. J. Biol. Chem..

[128] Wei P., Gao S., Han G. (2025). Evidence for Genetic Causal Association Between the Gut Microbiome, Derived Metabolites, and Age-Related Macular Degeneration: A Mediation Mendelian Randomization Analysis. Biomedicines.

[129] Page M.J., Kell D.B., Pretorius E. (2022). The Role of Lipopolysaccharide-Induced Cell Signalling in Chronic Inflammation. Chronic Stress.

[130] Tsioti I., Steiner B.L., Escher P., Zinkernagel M.S., Benz P.M., Kokona D. (2023). Endothelial Toll-like receptor 4 is required for microglia activation in the murine retina after systemic lipopolysaccharide exposure. J. Neuroinflamm..

[131] Noailles A., Maneu V., Campello L., Lax P., Cuenca N. (2018). Systemic inflammation induced by lipopolysaccharide aggravates inherited retinal dystrophy. Cell Death Dis..

[132] Leung K.W., Barnstable C.J., Tombran-Tink J. (2009). Bacterial endotoxin activates retinal pigment epithelial cells and induces their degeneration through IL-6 and IL-8 autocrine signaling. Mol. Immunol..

[133] Feng Y.F., Guo H., Yuan F., Shen M.Q. (2015). Lipopolysaccharide Promotes Choroidal Neovascularization by Up-Regulation of CXCR4 and CXCR7 Expression in Choroid Endothelial Cell. PLoS ONE.

[134] Oveson B.C., Iwase T., Hackett S.F., Lee S.Y., Usui S., Sedlak T.W., Snyder S.H., Campochiaro P.A., Sung J.U. (2011). Constituents of bile, bilirubin and TUDCA, protect against oxidative stress-induced retinal degeneration. J. Neurochem..

[135] Larsen P.P., Feart C., Pais de Barros J.P., Merle B.M.J., Gayraud L., Delyfer M.N., Korobelnik J.F., Delcourt C. (2023). Association of Age-Related Macular Degeneration with a Blood Biomarker of Lipopolysaccharide, a Gut Bacterial Proinflammatory Toxin. Investig. Ophthalmol. Vis. Sci..

[136] Ridlon J.M., Kang D.J., Hylemon P.B. (2006). Bile salt biotransformations by human intestinal bacteria. J. Lipid Res..

[137] Warden C., Barnett J.M., Brantley M.A. (2020). Taurocholic acid inhibits features of age-related macular degeneration in vitro. Exp. Eye Res..

[138] Zhou B., Parekh Z., Phung C., Rodriguez S.H., Skondra D. (2026). The gut-retina axis in age-related macular degeneration: Immune crosstalk and metabolite production. Exp. Biol. Med..

[139] Hammond C.L., Roztocil E., Gupta V., Feldon S.E., Woeller C.F. (2022). More than Meets the Eye: The Aryl Hydrocarbon Receptor is an Environmental Sensor, Physiological Regulator and a Therapeutic Target in Ocular Disease. Front. Toxicol..

[140] Dundar A., Cetik Yildiz S. (2025). Kynurenine pathway metabolites as potential biomarkers in age-related macular degeneration: An ELISA-based prospective study. Int. J. Retin. Vitr..

[141] Vilkeviciute-Petraite A., Bruzaite A., Cebatoriene D., Zaliuniene D., Lukosevicius R., Skieceviciene J., Kupcinskas J., Liutkeviciene R. (2026). The Role of Kynurenine and 5-Hydroxytryptophan in Modulating Microbiota and Their Implications in Exudative Age-Related Macular Degeneration. Diagnostics.

[142] Navratil E.M., Liu X., Wiley L.A., Anderson M.G., Meyer K.J., Brown R.F., Evan I.A., Taylor E.B., Stone E.M., Tucker B.A. (2026). Metabolic Analysis of Human Retinal Pigment Epithelium and Choroid Tissue in Aging and Macular Degeneration. bioRxiv.

[143] Sun X., Jiao X., Ma Y., Liu Y., Zhang L., He Y., Chen Y. (2016). Trimethylamine N-oxide induces inflammation and endothelial dysfunction in human umbilical vein endothelial cells via activating ROS-TXNIP-NLRP3 inflammasome. Biochem. Biophys. Res. Commun..

[144] Seldin M.M., Meng Y., Qi H., Zhu W., Wang Z., Hazen S.L., Lusis A.J., Shih D.M. (2016). Trimethylamine N-Oxide Promotes Vascular Inflammation Through Signaling of Mitogen-Activated Protein Kinase and Nuclear Factor-kappaB. J. Am. Heart Assoc..

[145] Zhou Y., Wang Z., Huang C., Yu X., Cai X., Zhao D., Cai Y. (2025). Genetically predicted causal links between gut microbiota and biological aging phenotypes in age-related macular degeneration. Biogerontology.

[146] Zhou Y., Liu J., Wang Z., Huang C., Wang Q., Yu X., Dai C., Zhao D., Cai Y., Wang T. (2025). Biological aging phenotypes mediate gut microbiota effects on age-related macular degeneration subtype progression: Genetic causality by mendelian randomization and mediation analysis. AMB Express.

[147] Yu J., Yuan J. (2025). Association between gut microbiota dysbiosis and age-related macular degeneration progression: A bioinformatics approach. Exp. Eye Res..

